# Surgical repair of thoracoabdominal aortic aneurysms using the critical artery reattachment technique

**DOI:** 10.1016/S1674-8301(11)60029-8

**Published:** 2011-05

**Authors:** Yulong Hou, Jianqiang Zhao, Wei Guo, Su Huang, Chunling Wang

**Affiliations:** a Department of Cardiothoracic Surgery, and; b Department of Hematology, Huai'an First Hospital Affiliated with Nanjing Medical University, Huai'an, Jiangsu 223300, China.

**Keywords:** thoracoabdominal aortic aneurysm, vascular graft replacement

## Abstract

In the study, we sought to retrospectively analyze the effectiveness and safety of surgical repair of thoracoabdominal aortic aneurysm using the critical artery reattachment technique. Twenty-three consecutive thoracoabdominal aortic aneurysm patients were treated using the technique of sequential aortic clamping and critical artery reattachment. The entire procedure was technically successful in all patients. One died of renal failure and the overall hospital mortality was 4.35%. The total incidence of complications was 21.74%. At a median follow-up of 33 months, all patients were alive. We found that the application of critical artery reattachment technique in the management of thoracoabdominal aortic aneurysm provides excellent short- and mid-term results in most patients. It could markedly increase the curing rate and reduce the morbidity of postoperative complications including paraplegia, ischemia of abdominal viscera, and renal failure.

## INTRODUCTION

Thoracoabdominal aortic aneurysm refers to descending thoracic or infrarenal abdominal aortic aneurysms. The most serious complications of aortic aneurysms are aortic dissection and rupture, which often result in the death of the patient. With the ageing of the population and the improvement of diagnostic capabilities, more and more patients are increasingly being referred for treatment of thoracoabdominal aortic aneurysm. The conventional open repair of thoracoabdominal aneurysms and dissections remains complex and demanding and is associated with significant morbidity and mortality[1]–[4]. Improving the therapeutic effect of surgery requires continuous improvement of surgical methods. In the present paper, we sought to retrospectively analyze the effectiveness and safety of surgical treatment in 23 cases of thoracoabdominal aortic aneurysm using the technique of sequential aortic clamping and critical artery reattachment.

## MATERIALS AND METHODS

### Patients

From February 2006 to December 2009, we treated 23 consecutive patients with thoracoabdominal aortic aneurysm at the Department of Cardiothoracic Surgery of Huai'an First Hospital affiliated with Nanjing Medical University, Huai'an, Jiangsu, China. The extent of thoracoabdominal aortic aneurysm was defined by the Crawford classification[5]. The diagnosis and extent of thoracoabdominal aortic aneurysm in all patients were confirmed by vessel Doppler ultrasonography and a 64-section computed tomographic angiography (CTA)[2],[5],[6].

### Surgical procedure

All surgeries were performed with the left femoro-femoral bypass with full heparinization. We consistently administered intravenous heparin (1.0 mg/kg) before aortic clamping. We tried to maintain the blood pressure of the upper limb at or above 80 mmHg and the perfusion pressure above 70 mmHg by adjusting the perfusion volume of the left femoral artery. Visceral and renal perfusion was carried out in conjunction with distal aortic perfusion. Each flow depended upon visceral or renal vessel resistance, and the median flow was 160 mL/min (range 85-225 mL/min). Blood flow was considered appropriate when the patient's urine output was 0.5 mL/min. We routinely allowed the rectal temperature to drift down to a target range of 32°C to 34°C. After the repair was completed, we irrigated the operative field with warm saline to reverse cooling. Cerebrospinal fluid (CSF) pressure was assessed with an intrathecal catheter and maintained during the procedure and in the intensive care unit until the third postoperative day. CSF was allowed to drain spontaneously if pressure increased above 10 mmHg.

The location and extent of lesion of thoracoabdominal aortic aneurysm in 23 patients involved the celiac artery origin and/or other critical arteries. During these open operations, we used the critical artery reattachment and sequential aortic clamping technique in order to maximize organ protection.

Intubation was performed with a double-lumen endotracheal tube, enabling collapse of the left lung. Patients were placed in a left helical position on a vacuum beanbag. The thoracoabdominal aortic aneurysm was exposed along the extraperitoneal plane using a left thoracolaparotomy through the seventh intercostal space. When aneurysm contours were completed, a femoro-femoral bypass was started with full heparinization.

In general, aortic reconstruction was performed from proximal to distal. A clamp was placed proximal to the aneurysm (between the left common carotid and left subclavian artery in type I or type II aneurysm). The aorta was opened longitudinally and divided circumferentially a few centimeters beyond the proximal clamp and anastomosed proximally in an end-to-end fashion to a woven graft using polypropylene suture. While the proximal end was being anastomosed, segmental, visceral and renal arteries were perfused by placing a distal clamp at the mid-thoracic level. After proximal anastomosis was completed, the aortic clamp was repositioned onto the vascular graft, and flow was restored to the left subclavian artery. The distal clamp was then moved to the supra celiac level, and patent intercostals arteries in this region were sewn over. Patent lower intercostal arteries (including segmental arteries below the T8 to L2 level) were reattached to an opening in the graft. After implantation of the segmental arteries, the proximal clamp was moved below those, allowing perfusion of the lower intercostals and lumbar arteries. If necessary, visceral and right renal arteries were reattached to an opening on the side of the graft. Finally, the left renal artery was also reattached. During reconstruction, balloon perfusion catheters were inserted into the celiac and superior mesenteric arteries to deliver selective visceral perfusion and a cold renal delivery system provided selective renal hypothermia. After completion of the anastomosis of the visceral and renal arteries, the balloon cannulas were removed from the origins of those arteries, and sequential aortic clamping was used to restore visceral, right renal perfusion and left renal perfusion. The distal end of the graft was sutured to the distal arteries. Whenever possible, the phrenic, vagus, and recurrent laryngeal nerves were preserved during the repair.

## RESULTS

### Patient demographic and disease characteristics

**Fig.1:**
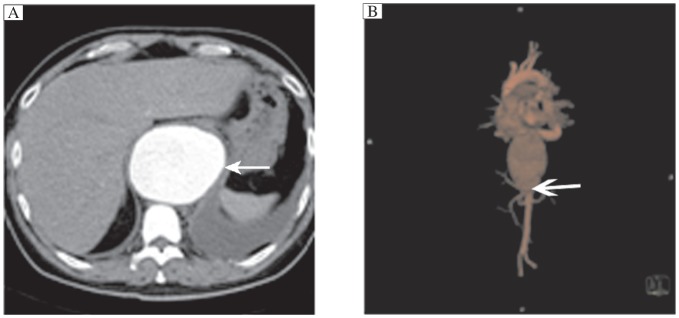
CT angiography image of thoracoabdominal aortic aneurysm. A: The diameter of the aneurysms was 11.0 cm (arrow). B: The lesion involves the celiac artery.

Twenty-three patients with thoracoabdominal aortic aneurysm were included in the current analysis. There were 15 males and 8 females. Their median age was 61.5 years (range 33-76 years). Six patients were asymptomatic and their thoracoabdominal aortic aneurysm was discovered incidentally. Seventeen patients presented with symptoms such as severe chest, abdomen, or back pain, thoracic cavity bleeding, dysphagia, or weight loss. The mean maximal diameter of the aneurysm was 6.7 cm (range 4.8-12.2 cm, ***Fig. 1***). Eight patients had type I, 5 had type II aneurysm, 7 had type III, and 3 had type IV aneurysm.

### Surgical outcomes

The median operation time for thoracoabdominal aortic aneurysm patients was 418 min (range 161-720 min). The average cross clamp duration of the patients was 151 min (range 76-538 min), the average duration of the left femoro-femora bypass was 192 min (range 70-493 min). The entire procedure was technically successful in all patients. One patient had suffered from renal insufficiency before the surgery due to aortic dissection and subsequent renal artery compression, and encountered secondary renal failure and respiratory failure after the procedure. The patient who underwent haemodialysis after surgery died on the 18^th^ postoperative day subsequent to multiorgan failure. In-hospital mortality was 4.35% (1 patient). Major postoperative complications like paraplegia, stroke and myocardial infarction did not occur. Two patients (8.69%) developed pneumonia and one patient (4.35%) showed evidence of respiratory distress syndrome. All three patients required prolonged (>48 h) ventilatory support. The median intubation time was 1.7 d (range 0.37-28 d). One patient (4.35%) was returned to the operation room due to postoperative bleeding. One patient (4.35%) had thoracic cavity effusion. The total incidence of complications was 21.74%. The mean pre-operative creatinine level was 128 µmol/L, which reached a mean maximal level of 158 µmol/L and returned to 94 µmol/L at discharge. The creatinine level at discharge was not significantly different from the preoperative level (*P* > 0.05). The patients were discharged at a median of 16 d. At a median follow-up of 33 months (4-49 months), all patients (14 men and 8 women) were alive. Surveillance CT revealed no new or false aneurysms.

## DISCUSSION

The natural history of thoracoabdominal aortic aneurysm shows poor prognosis as illustrated by high dissection rates in non-operated patients. In addition, the ageing population and improvement in diagnostic capabilities have caused a substantial increase of surgical demand by thoracoabdominal aortic aneurysm patients. However, open surgical treatment of thoracoabdominal aortic aneurysm remains to be associated with significant mortality[1]–[4], and there is also a significant risk of visceral, renal and spinal cord ischemia[2]. To reduce the considerable operative morbidity and mortality, investigators have devised many strategies to substantially improve the surgical outcome of thoracoabdominal aortic aneurysm. With the application of new strategies to further reduce mortality and prevent end-organ ischemia associated with thoracoabdominal aortic aneurysm, surgical repair is widely considered as a first option for thoracoabdominal aortic aneurysm[8].

Despite surgical advances, thoracoabdominal aortic aneurysm patients remain at high risk for the development of postoperative complications. Before operation, the risk of rupture has to be weighed against the risk of an adverse surgical outcome. Attentive selection of patients for open surgical repair is based on extensive operative risk assessment. Continuous improvement of perioperative management of patients and application of new surgical techniques is essential to reduce the incidence of complications.

Preoperative management of patients undergoing thoracoabdominal aortic aneurysm repair has evolved substantially during the past two decades. Several authors have reported excellent results using different protective strategies such as motor evoked potential monitoring, epidural cooling, and hypothermic circulatory arrest[3],[9],[10]. Our application of critical artery reattachment and sequential clamping technique on the surgical therapy of thoracoabdominal aortic aneurysm provides excellent results. The surgical cure rate was 95.65%. Routine surgical modalities include use of moderate systemic heparinization, mild permissive hypothermia (32°C to 34°C) and sequential aortic clamping. Our current strategy to prevent organ damage in elective patients consists of the use of distal aortic perfusion (keeping the distal perfusion pressure at or higher than 70 mmHg) in combination with liberal reattachment of critical arteries, cerebrospinal fluid drainage, cold crystalloid renal perfusion, and selective visceral perfusion. This multimodality approach is based on findings of other surgeons with vast experiences[11] and is also supported by our own results.

In sequential clamping technique, as the aorta is replaced from the proximal to the distal extent of the lesion, the aortic clamp is moved sequentially to lower positions along the graft to restore perfusion to newly reattached branch vessels. Sequential clamping of the aorta can reduce ischemic time and improve abdominal viscera perfusion. In theory, the reattachment of critical arteries should prevent spinal cord injury by preserving circulation to the anterior spinal artery; however, the issue of which specific arteries to ligate and which to reattach remains in dispute. Although Griepp *et al.*[12] considered that the spinal cord has redundant blood supply according to the collateral network theory and questioned the usefulness of critical artery reattachment, we and others[13] believed that because of the anatomic complexities of the spinal cord circulation, and the commonly incomplete anterior spinal artery, as well as individual anatomic variations among patients, aggressive reattachment strategies, especially those between T8 and L2, can prevent spinal cord problems. Although clinically significant postoperative manifestations of hepatic, pancreatic, and bowel ischemia are infrequent, they can have devastating impact when they occur[14]. To reduce the risk of perioperative coagulopathy and bacterial translocation, after the aorta was opened adjacent to the visceral branches, we used separate balloon perfusion catheters to selectively perfuse the visceral arteries. Passive hypothermia was used in all cases. We intentionally allowed the body temperature to drop to 32°C-34°C during the procedure. Hypothermia, by decreasing the metabolic demands of the spinal cord, may be beneficial in preventing postoperative paraplegia. At the completion of the operation, patients were rewarmed to 37°C. Our experience with regard to CSF drainage during the repair of thoracoabdominal aortic aneurysm is that the amount of CSF drained is dependent on the CSF pressure, which has to be kept below 10 mmHg. Postoperative renal dysfunction is another major concern, so we carefully monitor urine output and serum creatinine levels throughout the postoperative period. Intravenous antibiotics are continued until all drains, chest tubes, and central venous lines are removed.

In summary, our experience showed that the use of critical aortic clamping and segmental artery reattachment technique could markedly improve the curing rate and reduce the morbidity of postoperative complications including paraplegia, ischemia of abdominal viscera, and renal failure.
